# In This Issue

**Published:** 2002

## INTERNATIONAL GENDER AND ALCOHOL RESEARCH: RECENT FINDINGS AND FUTURE DIRECTIONS

Gender’s influence on drinking patterns and alcohol-related problems has attracted research attention on an increasingly international scale in recent years. One such research effort, an international collaborative research project called the International Research Group on Gender and Alcohol (IRGGA), was designed to coordinate international studies on women and alcohol and to devise new means of facilitating comparisons on gender and alcohol use in a wide range of countries. Drs. Sharon C. Wilsnack and Richard W. Wilsnack briefly summarize IRGGA’s work, identifying future directions for research in this area and suggesting the possible implications of this research for developing more gender-sensitive national and international alcohol policy. (pp. 245–250)

## MINORITY WOMEN AND ALCOHOL USE

Does belonging to a particular ethnic or racial group influence a woman’s tendency to drink? Drs. R. Lorraine Collins and Lily D. McNair explore women’s drinking patterns in four main U.S. minority groups: African Americans, Asian Americans, Latinas, and American Indians. The authors examine how drinking patterns among these groups are influenced by specific characteristics of each group—including religious activity; genetic risk/protective factors; level of acculturation to U.S. society; and historical, social, and policy variables. In many cases, these factors tend to protect women from developing problems with alcohol. The authors point out, however, that a great deal of variability exists within each minority group in addition to commonalities, and additional information is needed to better understand the unique role that ethnicity plays in a woman’s likelihood to drink. (pp. 251–256)

## INFLUENCE OF ALCOHOL AND GENDER ON IMMUNE RESPONSE

Women tend to have hardier immune systems than men, especially during their reproductive years, apparently because women’s high levels of estrogen help stimulate immunity and fight disease. Alcohol use dramatically compromises immune responses. It is unknown, however, if gender differences exist in how alcohol affects those responses. In this article, Drs. Elizabeth J. Kovacs and Kelly A.N. Messingham review research suggesting that chronic heavy drinking (but not lighter drinking) depresses estrogen levels, nullifying estrogen’s beneficial effects on the immune system, and weakening a woman’s ability to fight infections and tumors. Some research suggests that this detrimental effect may be compounded by an alcohol-induced elevation in steroidal hormones, known as glucocorticoids, which suppress immune responses in both men and women. (pp. 257–263)

## SEX DIFFERENCES IN THE GENETIC RISK FOR ALCOHOLISM

Men and women differ in their risk for alcoholism, and these sex-based differences may be at least partially rooted in genetics. Dr. Carol A. Prescott reviews findings from studies of families with alcoholic members, suggesting that the extent to which risk for alcoholism is inherited is greater among men than among women. However, the findings vary greatly among the studies, preventing definite conclusions. Genetic factors related to risk of alcoholism—or the patterns of transmitting these risk factors—could differ for men and women. The author cautions, however, that current research on sex differences has limitations that must be overcome before the findings can be generalized and the potential applications of such research can be fully realized. (pp. 264–273)

## ALCOHOL’S EFFECTS ON FEMALE REPRODUCTIVE FUNCTION

Mild-to-moderate alcohol use affects female reproductive function throughout the life cycle. Animal studies show that alcohol consumption disrupts female puberty, and drinking during this period also may influence growth and bone health. After puberty, alcohol also has been found to disrupt normal menstrual cycling and reproductive function and to affect important hormone levels in postmenopausal women. Alcohol use also can have implications for bone health in adult women. Drs. Mary Ann Emanuele, Frederick Wezeman, and Nicholas V. Emanuele describe normal female reproduction, including puberty, the normal female cycle, and hormonal changes in postmenopausal females. The authors then discuss research into the effects of alcohol on these key events in female reproductive function. (pp. 274–281)

## EFFECTS OF PRENATAL ALCOHOL EXPOSURE ON CHILD DEVELOPMENT

Children prenatally exposed to alcohol may exhibit a number of developmental deficits. The terms applied to these children vary depending on the severity of the deficits; they include fetal alcohol syndrome, fetal alcohol effects, alcohol-related birth defects, and alcohol-related neurodevelopmental disorder. Drs. Joseph L. Jacobson and Sandra W. Jacobson review these conditions and present research findings on the cognitive and behavioral effects of prenatal exposure to alcohol, which include hyperactivity and problems with attention, learning, memory, and social and emotional development. (pp. 282–286)

## ALCOHOL’S EFFECTS ON ADOLESCENTS

Research on alcohol’s effects on the developing adolescent is still in its infancy, despite the fact that many young people begin drinking during adolescence. Evidence exists that people who begin drinking at an early age are more likely to have problems with alcohol later in life. Research also has shown that adolescence is a time when remarkable changes are taking place in the brain. Dr. Linda Patia Spear examines findings on alcohol’s effects in adolescents, with special emphasis on the impact of alcohol on neural and endocrine development. Though the research in this area is scarce, gender-specific effects are highlighted whenever possible. (pp. 287–291)

## ALCOHOL AND OTHER FACTORS AFFECTING OSTEOPOROSIS RISK IN WOMEN

Women reach their peak bone mass by about age 35. After that age, a woman’s bone mass slowly declines until after menopause, when the loss becomes more rapid. For this reason, women must take steps to build strong bone early in life, and one of those steps should be to monitor alcohol consumption. Dr. H. Wayne Sampson describes studies in both humans and other animals that indicate that chronic alcohol consumption compromises bone health, resulting in a weakening of the bones’ mechanical structure. The effect of moderate drinking on bone health is less clear. Some research in humans indicates that moderate drinking may boost bone mass, whereas animal studies have contradicted this finding. The author also briefly reviews other lifestyle factors, such as tobacco use, nutrition, weight-bearing exercise, body weight, and hormone replacement therapy, all of which influence bone development. Additional research is needed to determine how those factors may interact with alcohol to affect bone health. (pp. 292–298)

## HEALTH ISSUES IN POSTMENOPAUSAL WOMEN WHO DRINK

Light-to-moderate alcohol consumption has been shown to increase women’s estrogen levels, which may help guard against the development of coronary heart disease and osteoporosis. Conversely, a number of epidemiological studies indicate that even moderate alcohol consumption is associated with increased breast cancer risk. The reasons for this effect are not clearly understood. Drs. Thomas C. Register, J. Mark Cline, and Carol A. Shively review the effects of alcohol consumption on these three diseases in postmenopausal women. The authors stress that the data on alcohol’s potential health effects in postmenopausal women should be interpreted carefully, given the diversity that is found within nondrinking control groups, the inaccuracies of self-report, and the lack of studies in which subjects are randomly assigned to drinking conditions. (pp. 299–307)

## USE AND MISUSE OF ALCOHOL AMONG OLDER WOMEN

Older women may be at particular risk for alcohol-related problems. They are more likely than men to outlive their spouses and to face other losses that typically lead to loneliness and depression—factors that may prompt alcohol use. As Drs. Frederic C. Blow and Kristen Lawton Barry explain, women also are physiologically at greater risk for alcohol-related health problems as they age. Because of these risks, alcohol use recommendations for older women generally are lower than those set for both older men and younger women. Potential alcohol problems can be detected by routine screening in primary health care settings, clinics, nursing homes, and other facilities. The use of screening and brief intervention may help minimize alcohol problems in older women. Although research on the success of brief interventions with this population is limited, the findings are promising. (pp. 308–315)

## NEURODEGENERATION IN WOMEN

Are women who drink more likely to suffer from Alzheimer’s disease (AD) or a similar disorder? In this short article, Dr. Farida Sohrabji reviews evidence that, compared with men, women face greater risk for neurodegenerative diseases such as AD because of the loss of key hormones at menopause. The author discusses the evidence that heavy alcohol consumption may increase the risk for AD in both sexes but especially in females, and describes experimental research designed to determine whether females are more vulnerable than males to alcohol-induced neurocognitive effects. (pp. 316–318)

